# ﻿A new species of *Pseudodiaptomus* (Copepoda, Calanoida, Pseudodiaptomidae) from the Great Barrier Reef, Australia

**DOI:** 10.3897/zookeys.1261.152830

**Published:** 2025-11-21

**Authors:** Julian Uribe-Palomino, T. Chad Walter

**Affiliations:** 1 Commonwealth Scientific and Industrial Research Organisation (CSIRO) Environment, Queensland Biosciences Precinct, Brisbane, Australia Commonwealth Scientific and Industrial Research Organisation (CSIRO) Environment, Queensland Biosciences Precinct Brisbane Australia; 2 National Museum of Natural History, Smithsonian Institution, Washington, DC, USA National Museum of Natural History, Smithsonian Institution Washington United States of America

**Keywords:** Copepods, demersal, endemic species, South Pacific Ocean

## Abstract

A new species of the demersal calanoid copepod genus *Pseudodiaptomus* is described from specimens collected from the Great Barrier Reef, Australia. This species most closely resembles *P. burckhardti* in the structure of male fifth leg with reduced endopods on both legs, but differs from the latter in several features, including armatures of male fifth leg, asymmetrical female urosome and posterodorsal projections on the male prosome.

## ﻿Introduction

Copepods are highly diverse micro-crustaceans and are probably the most abundant of the metazoans in aquatic systems. Their habitats range from free-living planktonic, meiobenthic, demersal and even parasitic. The free-living copepods of the genus *Pseudodiaptomus* Herrick, 1884 currently, consists of 84 valid species (Walter and Boxshall 2025). Members of this genus are primarily demersal and typically found in shallow coastal estuarine-marine environments, extending from freshwater to hypersaline waters, and reside near or on the bottom during the day and migrate throughout the water column from dusk to dawn (Walter 1984, 1986, 1987).

The mouthparts and swimming legs are very similar among the species, making these body parts of limited use for species determination even though, it is known some variability among the *Pseudodiaptomus* species (Walter 1986; Srinui et al. 2013; Mulyadi 2022; Nishida et al. 2024). However, the fifth pair of male swimming legs are highly dimorphic and have high taxonomic value, and occasionally in females the prosome and urosome augmentations are species specific. The separation of species in this genus is facilitated by using species groups and subgroups (Walter 1986), which were updated by Walter et al. (2002: 666).

From the 84 accepted species of *Pseudodiaptomus*, it is known that 16 species of them inhabit coastal and surrounding waters from Australia, which has a vast territory in the Pacific, the Indian, and the Southern Oceans mainly, but also includes maritime and insular territory in tropical equatorial waters. Those species and their primary location are mentioned as follow: *P. annandalei*Sewell (1919) [Queensland]; *P. australiensis*Walter (1987) [Northern Territory]; *P. baylyi*Walter (1984) [Northern Territory, Queensland]; *P. clevei* Scott A. (1909) [Aru Island, Papua New Guinea, Australia]; *P. colefaxi*Bayly (1966) [Queensland, New South Wales, Victoria]; *P. cornutus*Nicholls (1944) [Victoria, South Australia] ; *P. galleti* (Rose, 1957) [Western Australia, Shark Bay]; *P. griggae*Walter (1987) [Papua New Guinea, Aru Archipelago and Queensland]; *P. hickmani*Sewell (1912) [New South Wales, Cooks River]; *P. hypersalinus*Walter (1987) [Western Australia, Shark Bay], *P. inflexus*Walter (1987) [Queensland]; *P. mertoni*Früchtl (1923) [Aru Islands, Indonesia; Queensland, Port Curtis]; *P. occidentalus*Walter (1987) [Western Australia]; *P. ornatus* (Rose, 1957); *P. pacificus*Walter (1986) [Queensland]; *P. serricaudatus* (Scott T., 1894) [Queensland, Heron Island].

The new species described in this paper is based on material found in samples from the Great Barrier Reef that were donated by A.D. McKinnon and M.S. Talbot during 1978–1980. These collections have been housed at the Smithsonian Institution, National Museum of Natural History (NMNH) for the last 40 years and only recently studied and determined that they contained this new species.

## ﻿Materials and methods

Specimens were fixed and preserved in 10% neutralized formalin/seawater after collection and then were transferred to 70% ethanol at the Smithsonian Institution, United States National Museum (USNM). Male and female specimens were stained with chlorazol black and transferred to depression slides in a solution of glycerine–ethanol for detailed examination under a Wild stereoscope. Habitus and dissected appendages were examined and illustrated with the aid of a camera lucida attached to a Nikon Optiphot DIC Phase Contrast Compound Microscope. Digitalization of the drawings was achieved by using Adobe Illustrator v. 27.9.6. Z-stacking images of the habitus of a pair of adult male and female specimens and their corresponding fifth legs were obtained using an Olympus DP80 digital camera attached to an automated upright epifluorescence microscope Olympus BX63 with CellSens operating software and a motorized Z-drive from the USNM. Body measurements of male and female specimens were made using a fluorescence microscope Olympus BX51 also at USNM.

All measurements were done with a calibrated ocular micrometre and are presented in millimetres. Prosome length was measured from its most anterior margin to the posterior tip of the prosome and urosome length was measured from the anterior insertion on prosome to the posterior tip of caudal rami.

Body and appendage terminology follows the terminology established by Huys and Boxshall (1991).

## ﻿Systematics

**Order Calanoida Sars, 1903**

**Family Pseudodiaptomidae Sars, 1902**

**Genus *Pseudodiaptomus* Herrick, 1884**

### ﻿ Pseudodiaptomus greenwoodi sp. nov.

Taxon classificationAnimaliaCalanoidaPseudodiaptomidae

1F8DFBFF-73CC-57AC-B14E-4689073140EE

https://zoobank.org/D8C6AF0B-75AE-4C68-B35F-6B102D077413

Figs 1, 2, 3, 4, 5

#### Type locality.

Australia, Queensland, Great Barrier Reef, Lizard Island, 14°40.45'S, 145°26.49'E, captured using light traps over coral reef.

#### Type specimens.

***Holotype***: ♂; USNM 1754446. ***Allotype***: ♀; USNM 1754447. ***Paratypes*: Australia** • 10 ♀♀, • 10 ♂♂; Great Barrier Reef, Lizard Island; Jan.1978; M.S. Talbot; USNM 1754448; specimens intact; all types removed from original collection USNM 1110847.

#### Additional material.

**Australia** • 2 ♂♂, 2 ♀♀; Great Barrier Reef; Jul. 2000; A.D. McKinnon; **USNM** 310675 • 3 ♀♀; Queensland, Haughton River Estuary; Sep 1992; A.D. McKinnon; USNM 310761 • 100+ specimens; Queensland, Great Barrier Reef, coral reef, Lizard Island; Jan. 1977–Jan. 1978; M.S. Talbot; USNM 1110845–1110847; captured using light traps over reefs.

#### Description.

**Figure 1.**
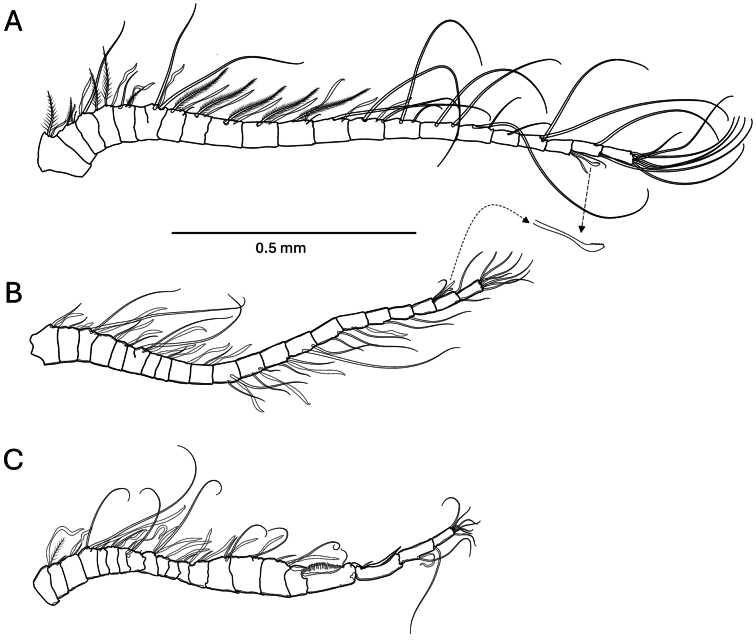
*Pseudodiaptomus greenwoodi* sp. nov. **A.** Female antennule; **B.** Male antennule left; **C.** Male antennule right.

Common features shared by females and males:

***Antennule*** (Fig. 1A, B) of females and left male antennule possessing 22 segments, segments 6 and 7 partly fused and counted separately, with modified barbed seta on segment 20.

Setal elements: (segment number = setae + aesthetasc [ae]) Female: **1** = 1 + ae, **2** = 2 + ae, **3** = 1 + ae, **4** = 2 + ae, **5** = 2 + ae, **6–7** = 2 + ae, **8** = 2 + ae, **9** = 1 + ae, **10** = 1 + ae, **11** = 1 + ae, **12** = 2 + ae, **13** = 1 + ae, **14** = 2 + ae, **15** = 2, **16** = 2, **17** = 2, **18** = 1, **19** = 2, **20** = 2 + ae, **21** = 2, **22** = 7. Male: **1** = 1 + ae, **2** = 1 + ae, **3** = 2 + ae, **4** = 1 + ae, **5** = 1 + ae, **6-7** = 2 + ae, **8** = 0 + ae, **9** = 1 + ae, **10** = 0 + ae, **11** = 1 + ae, **12** = 2 + ae, **13** = 2 + ae, **14** = 2 + ae, **15** = 2, **16** = 2, **17** = 1 + ae, **18** = 1, **19** = 1, **20** = 2 + ae, **21** = 2, **22** = 7.

**Figure 2.**
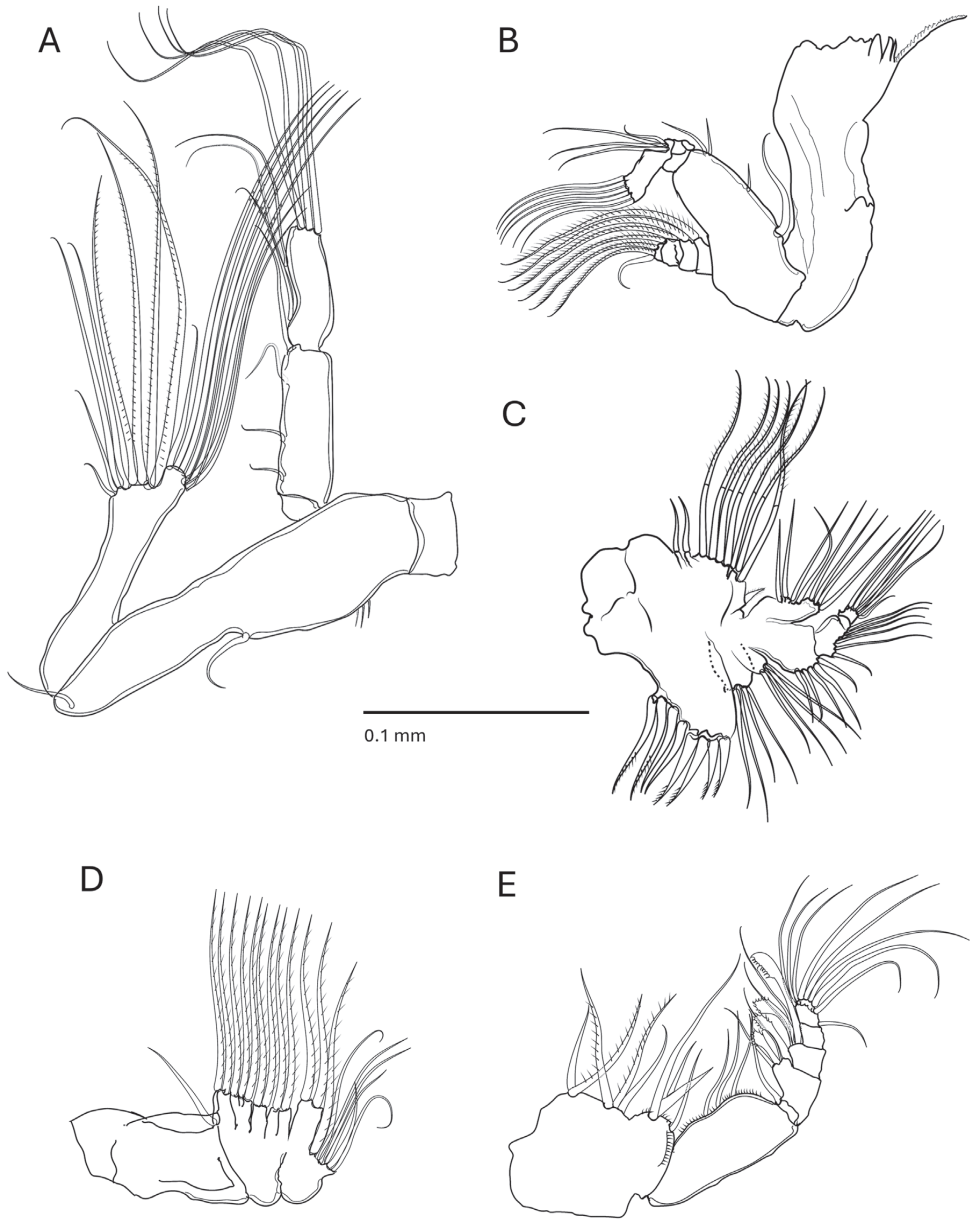
*Pseudodiaptomus greenwoodi* sp. nov., cephalic appendages. **A.** Antenna; **B.** Mandible; **C.** Maxillule; **D.** Maxilla; **E.** Maxilliped.

***Antenna*** (Fig. 2A): coxa without seta, basis and first endopodal segment completely fuse with 1 seta each, endopodal segment 2 with 8 and 8 setae on terminal and subterminal lobes respectively; exopod 3-segmented, first without seta, second with 1 proximal, 2 medial and 1 terminal setae; third with 3 medial and 4 terminal setae.

***Mandible*** (Fig. 2B): basis inner margin with 4 setae; endopod 2-segmented, first with 4 setae, second with 6 setae; exopod 5-segmented, first to fifth segments with 1, 1, 1, 1, 3 setae, respectively. Gnathobase with one long serrate dorsal seta and 3 cuspidate and 4 small, rounded teeth.

***Maxillule*** (Fig. 2C): praecoxal arthrite with 8 strong setae; coxa with 4 setae on endite and 9 on epipodite; basis with 4 and 5 setae on proximal and distal endites respectively; basal exite with 1 reduced seta; endopod 3-segmented, with 4, 2 and 6 setae respectively; exopod with 8 setae along margin.

***Maxilla*** (Fig. 2D): praecoxa, coxa and basis partially fused and, exopodite quite reduced without visible segmentation. Praecoxal endites with 3 and 3 setae respectively; coxal endites with 2 setae each; basal endite with 2 setae and endopod with 7 setae.

***Maxilliped*** (Fig. 2E): praecoxa and coxa completely fused, endites with 0, 2, 3, 4 setae; basis with 3 setae; endopod 6-segmented, with 1 seta on the first segment, second and third segments bearing 2 simple setae and 2 special robust mid-margin serrate setae; 1 seta on fourth and fifth segments and, 7 setae on sixth segment.

***Legs 1–4*** (not figured, as they are alike in shape and armature to figures published by Walter (1986), Srinui et al. (2013), and Nishida et al. (2024)), symmetrical and biramous with 3-segmented rami; coxa and basis of both rami with spinules on distal corner. Seta and spine formula as follows:

**Table:** 

	coxa	basis	exopod			endopod		
Leg 1	0-1	0-0	I-1;	0-1;	II, 1, 3	0-1;	0-1;	1, 2, 3
Leg 2–3	0-1	0-0	I-1;	I-1;	II, 1, 5	0-1;	0-2;	2, 2, 4
Leg 4	0-1	1-0	I-1;	I-1;	II, 1, 5	0-1;	0-2;	2, 2, 3

**Figure 3.**
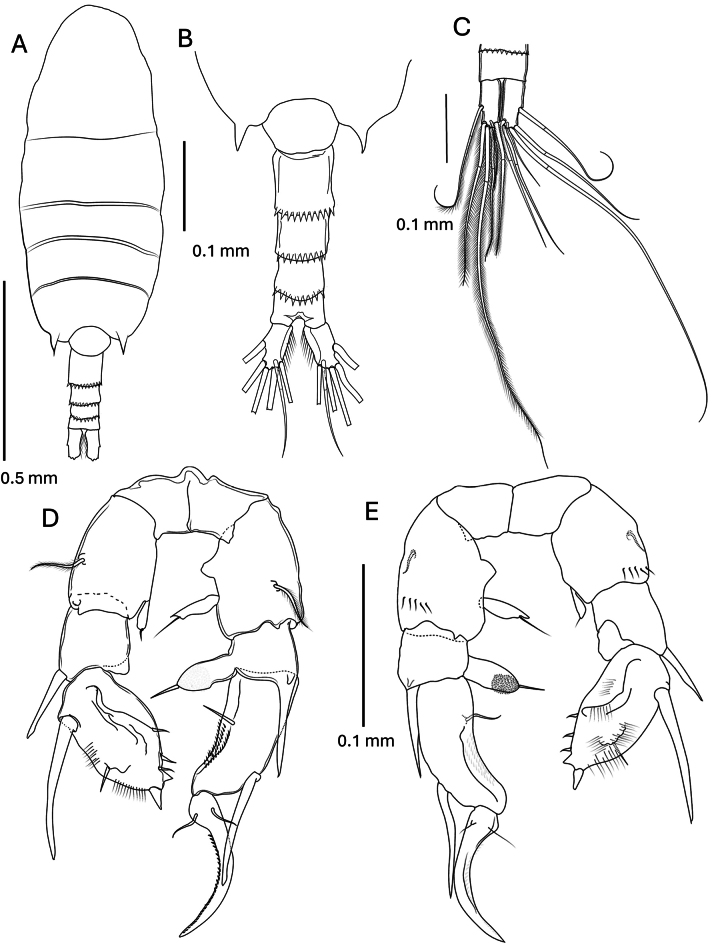
*Pseudodiaptomus greenwoodi* sp. nov., male. **A.** Habitus dorsal view; **B.** Urosome enlarged view; **C.** Caudal rami dorsal view; **D.** Leg 5 posterior view; **E.** Leg 5 anterior view.

**Male** (Figs 3A–E, 5C, D).

***Body*** length 1.11–1.12 mm (*n* = 5); prosome length 2.4 times urosome. Cephalosome and first pedigerous somite separate and pedigerous somites 4 and 5 fused with distolateral acute projections. Urosome 5-segmented, symmetrical, segments 2–4 posterodorsal margins with rows of acute spinule rows extending laterally. Caudal rami symmetrical, each ramus with 1 lateral, 4 terminal and 1 dorsal setae. Urosomites and caudal rami with proportions 12:27:14:12:14:21 = 100.

***Male right antennule*** (Fig. 1C) 21-segmented, segments 6 and 7 separate, geniculate at segment 18-19. Setal elements: **1** = 1 + ae, **2** = 1, **3** = 1 + ae, **4** = 2 + ae, **5** = 1, **6** = 2 + ae, **7**–**8** = 3 + ae, **9** = 1 + 2ae, **10** = 0 + ae, **11** = 0 + ae, **12** = 1 + ae, **13** = 1 + ae, **14** = 2 + ae, **15** = 1 + ae, **16** = 2 + ae, **17** = 0, **18** = 1, **19** = 1 and medial spiniform projection, **20** = 2 + ae, **21** = 7.

***Leg 5*** (Fig. 3D, E). Right leg: basis quadrate, with large plumose posterior-surface seta, anterior-surface sub-distal row of spinules, one small proximomedial knob-like process and a small digital medial endopod which is terminally pointed with short subterminal seta. Exopod 1 compressed, with distolateral large spiniform process, medial margin with large finger-like process terminally hirsute with fine spinules and terminal seta (visible in anterior view). Exopod 2 elongated and curved with a large distolateral spine reaching to midlength of exopod 3, medial margin with a groove lined with small spinules and a seta. Exopod 3 medially curved, tapering distally, medial margin with fine spinules and two proximal setae. Left leg: basis of similar size and shape to right leg basis with large lateral seta at mid-length, anterior-surface sub-distal row of spinules, and small distomedial endopod with terminal seta. Exopod 1 small quadrate with a large distolateral spine. Exopod 2 oval with a very large proximolateral spine, a small sub-lateral seta at mid-length, surrounded by fine spinules, terminates in a pair of short stout spines, the terminal spine larger, and 3 medial setae.

**Figure 4.**
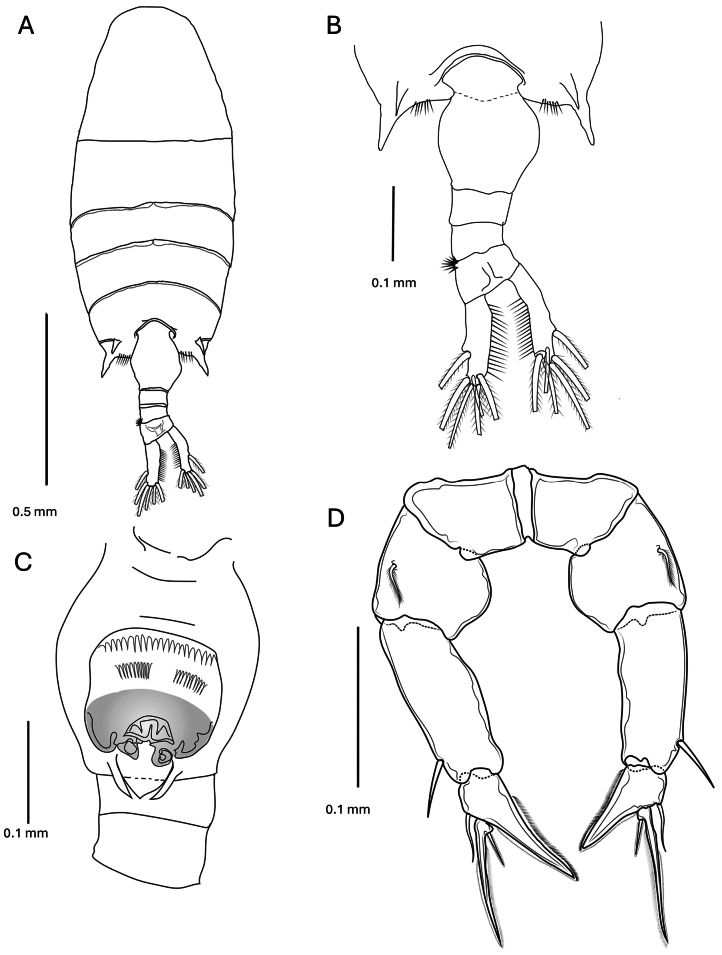
*Pseudodiaptomus greenwoodi* sp. nov., female. **A.** Habitus dorsal view; **B.** Urosome enlarged view; **C.** Urosome 1–3 ventral view; **D.** Leg 5 posterior view.

**Figure 5.**
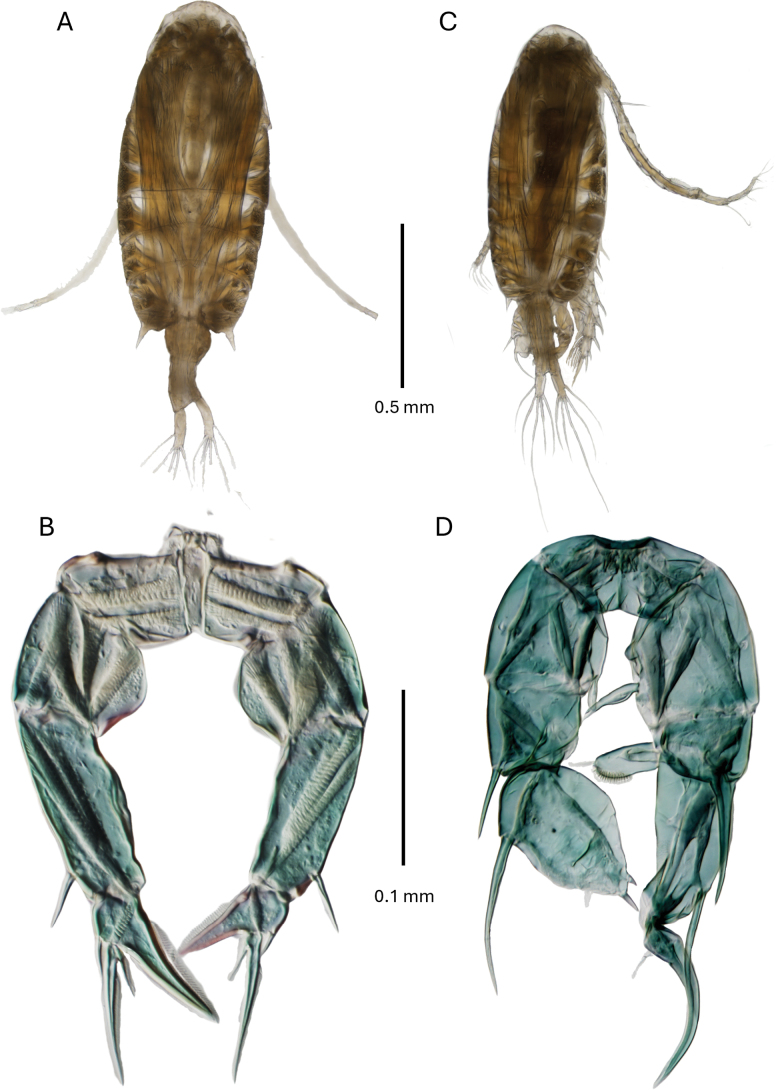
*Pseudodiaptomus greenwoodi* sp. nov., female. **A.** Habitus dorsal view; **B.** Leg 5 posterior view; male, **C.** Habitus dorsal view; **D.** Leg 5 posterior view.

**Female** (Figs 4A–D, 5A, B).

***Body*** length, 1.33–1.39 mm (*n* = 5). Prosome length 2.6 times urosome. Cephalosome and pedigerous somite 1 separate, pedigerous somites 4 and 5 completely fused with 2 symmetrical distally directed posterolateral spine-like projections and lined with fine spinules on each side of urosome insertion. Urosome 4-segmented, genital double-somite slightly asymmetrical, swollen at midlength and ventrally, darker towards the swollen genital aperture with double row of proximal spinules, distally the genital aperture is covered with a 3-lobed operculum, 2 lateral lobes thinner and expanded posteriorly, and a medial lobe triangulate tapering distally covering paired gonopores fused into a U-shaped structure. Urosomites 2 and 3 of similar size, lacking spinule rows on posterodorsal margins; somites 3 and 4 asymmetrical, right lateral margin reduced producing a curvature; somite 3 left posterolateral margin with small spinule patch. Caudal rami asymmetrical, right ramus mediolaterally curved and slightly thinner. Each ramus with typically 1 lateral, 4 terminal and a small dorsal setae. Medial margins of each ramus lined with fine setules. Urosomites 1–4 and caudal rami with proportions 34:12:12:13:29=100. The egg sac is single when present.

***Leg 5*** uniramous and slightly asymmetrical; basis slightly swollen medially, with a large plumose lateral seta on posterior surface. Exopods 3-segmented, exopod 1 rectangular and 2 times longer than basipod, with distolateral spine. Exopod 2 distomedial corner produced and pointed with serrated hyaline process, left one slightly larger than the right, and short distolateral seta. Exopod 3 slender with serrate margins and a proximomedial spine. Endopods lacking.

#### Etymology.

The species is named in honour of Dr Jack Greenwood (University of Queensland), an Australian copepod taxonomist, who donated many Australian pseudodiaptomid samples over the years to the second author.

#### Distribution.

This species is currently known from the north of the Great Barrier Reef up to coastal waters of Townsville and surrounding areas.

## ﻿Discussion

Morphological features of *Pseudodiaptomus greenwoodi* sp. nov. are unique among the Australian species with the female’s possession of paired posterior projections on pedigerous segments 5, and the male leg 5 left and right endopods are simple and much reduced in size. Based on the male leg 5 morphology, it is easy to assign this species to the Burckhardti species group of Walter (1986), as the leg 5 right and left endopods are rudimentary, small and simple, and exopod 1 has a small medial thumb shaped projection. The female and left male antennule have 22 segments and the 20^th^ a barbed seta, and females have a single egg sac.

**Figure 6.**
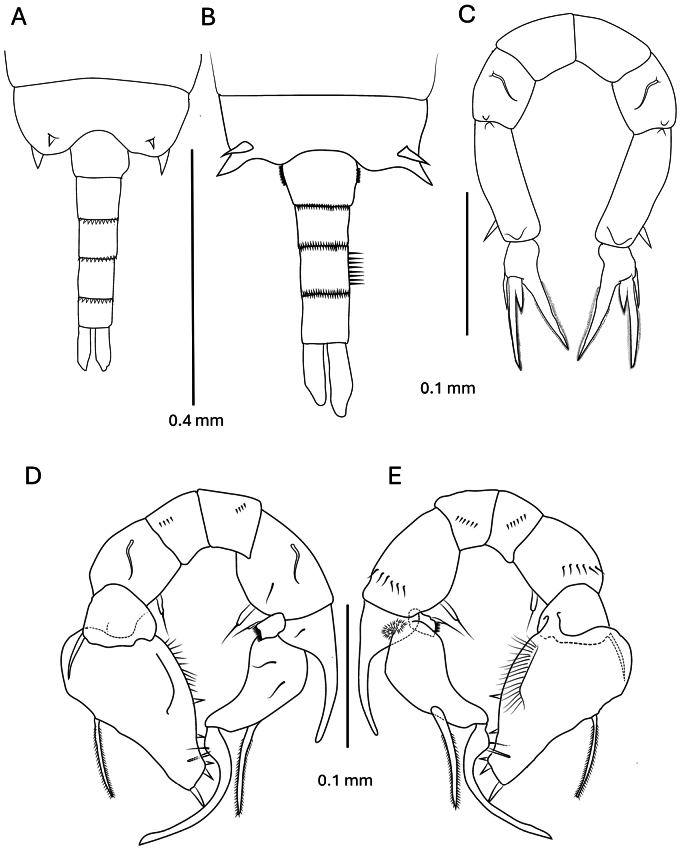
*Pseudodiaptomus burckhardti*Sewell (1932) redrawn from Walter (1986). **A.** Pedigerous somite 4–5 and urosome from male; **B.** Ibid., from female; **C.** Female leg 5 posterior view; **D.** Male leg 5 posterior view; **E.** Ibid., anterior view.

The original description of *P. burckhardti* Sewell, 1932 was based only on females collected from the Nicobar and Andaman Islands, and later Pillai (1980) redescribed the species and presented the male of the species from the same locality. His redescription did not report the lateral spinule row on the female urosomite 3, though he noted that the female leg 5 exopodal segment 1 distolateral corners with spinule patch. Pillai’s figures were very small and not clear. *Pseudodiaptomus burckhardti* was redescribed again by Walter (1986) from samples collected from Palau Islands. Male pedigerous somite with paired dorsal lateral projections (Fig. 6A), female urosomite 3 possesses a spinule row only on right lateral margin and simple leg 5 and asymmetrical caudal rami with the right ramus longer (Fig. 6B, C) furthermore, additional characters of the male right leg 5 were recognized such as, exopod segment 1 with a short proximomedial process, apex blunt and fringed with spinules and a terminal seta, the left exopod 2 large and proximally swollen narrowing towards apex with slender plumose spine at midlength (Fig. 6D, E).

*Pseudodiaptomus greenwoodi* sp. nov. differs from the former, in the following: female: pedigerous segment 5 with posterior spinule rows on each side of urosome insertion, urosomites 1–3 lack posterior spinule rows, urosomite 3 with spinule patch on left lateral margin only and the urosome and caudal rami are asymmetrical. Male: pedigerous segment 5 with single (not paired) distolateral projections and urosomite 2 the longest segment. Leg 5 is distinguished with: right exopod 1 medial margin process finger-like, distally rounded with terminal seta, left exopod 2 ovate with large strong proximolateral spine and very small lateral spine. The Burckhardti species group was a monotypic group before the addition of *P. greenwoodi* sp. nov

### ﻿A key of the Burckhardti species group:

**Table:** 

Female
1	Urosome and caudal rami are asymmetrical. Lack of posterior spinules on urosomites	***P. greenwoodi* sp. nov.**
2	Urosome and caudal rami symmetrical. Urosomite-3 bearing a row of lateral setae on right side	***P. burckhardti***
Male
1	Pedigerous somite-5 with single dorsolateral projections, leg 5 right exopod-1 medial margin with finger-like process, distally rounded, left exopod-2 ovate in shape with long strong proximolateral spine	***P. greenwoodi* sp. nov.**
2	Pedigerous somite-5 with paired dorsolateral projections, leg 5 right exopod-1 medial margin with blunt rectangular process, left exopod-2 triangular with a slender mediolateral spine	***P. burckhardti***

## Supplementary Material

XML Treatment for Pseudodiaptomus greenwoodi

## ﻿Additional information

### Use of AI

No use of AI was reported.

### Author ORCIDs

Julian Uribe-Palomino https://orcid.org/0000-0002-6867-2108

T. Chad Walter https://orcid.org/0000-0002-2368-9408

### Data availability

All of the data that support the findings of this study are available in the main text.
